# Retrospective validation of a rapid Lyme fluorescent immunoassay in differentiating Lyme arthritis from other musculoskeletal presentations in children in a Lyme-endemic region

**DOI:** 10.1128/spectrum.03593-23

**Published:** 2024-04-29

**Authors:** Alexis Donovan, Rebecca Quilty, Bryn K. Joy, Shahriar Seddigh, Heather Coatsworth, Luke Gauthier, Jeannette L. Comeau, Bianca Lang, Jason Leblanc, Todd Hatchette, Elizabeth Stringer

**Affiliations:** 1 Royal College of Surgeons in Ireland, Dublin, Ireland; 2 The Hospital for Sick Children, Toronto, Ontario, Canada; 3 Dalhousie University, Halifax, Nova Scotia, Canada; 4 Division of Orthopedic Surgery, Nova Scotia Health, Halifax, Canada; 5 National Microbiology Laboratory Branch, Public Health Agency of Canada, Winnipeg, Manitoba, Canada; 6 Division of Orthopedic Surgery, IWK Health, Halifax, Nova Scotia, Canada; 7 Division of Pediatric Infectious Diseases, IWK Health, Halifax, Nova Scotia, Canada; 8 Division of Pediatric Rheumatology, IWK Health, Halifax, Nova Scotia, Canada; 9 Department of Pathology and Laboratory Medicine, Nova Scotia Health, Halifax, Canada; University of Maryland School of Medicine, Baltimore, Maryland, USA

**Keywords:** Lyme disease, testing, Lyme arthritis, point of care, children, validation, *Borrelia*, *Borrelia burgdorferi*, serology, diagnosis

## Abstract

Lyme arthritis can present similarly to other causes of joint pain and swelling including septic arthritis and other acute and chronic arthropathies of childhood. Septic arthritis, although rare, constitutes an orthopedic emergency and requires early surgical intervention to reduce the risk of permanent joint damage. Currently, results of standard serologic tests to diagnose Lyme disease take days to weeks, which is unhelpful in acute clinical decision-making. Thus, some children with Lyme arthritis are treated empirically for septic arthritis undergoing unnecessary invasive procedures and hospital admission while on inappropriate antibiotic therapy. We retrospectively validated the Quidel Sofia Lyme Fluorescent Immunoassay, a rapid serologic assay that can detect IgG and/or IgM antibodies to *Borrelia burgdorferi* in 10 minutes, in residual serum samples collected from 51 children who had Lyme arthritis and 55 children with musculoskeletal presentations who were Lyme negative. The sensitivity and specificity of the Sofia IgG to identify cases of Lyme arthritis in children were 100% (95% confidence interval [CI] of 93.0%–100%) and 96.4% (95% CI: 87.5%–99.6%), respectively. The positive likelihood ratio (LR) was 27.5 (95% CI 7–107), and the negative LR was 0.00 (95% LR 0.00–0.15). We propose that the Sofia IgG, a rapid method for identifying Lyme arthritis, may be useful in differentiating Lyme arthritis from other forms of arthritis. Used in conjunction with readily available clinical and laboratory variables, it could help to rapidly identify children who are at low risk of septic arthritis in Lyme-endemic regions.

**IMPORTANCE:**

Lyme arthritis is a common manifestation of Lyme disease in children, with clinical features overlapping with other causes of acute and chronic joint pain/swelling in children. We have demonstrated that the Sofia IgG is a reliable test to rule in and rule out the diagnosis of Lyme arthritis in children with musculoskeletal presentations in a Lyme-endemic region. When used in conjunction with clinical and laboratory variables routinely considered when differentiating Lyme arthritis from other diagnoses, the Sofia IgG has the potential to fill an important gap in care, especially when acute decision-making is necessary. The Sofia IgG should be included in prospective research studies examining clinical prediction tools to identify children at low risk of septic arthritis.

## INTRODUCTION

Lyme disease (LD) is caused by the spirochete *Borrelia burgdorferi* and is transmitted to humans by the *Ixodes scapularis* tick in central and eastern Canada (1). Since it became a nationally reportable disease in 2009, LD has become a growing public health concern in Canada where the number of cases has continuously increased from 144 reported cases in 2009 to 3,147 cases in 2021 (2). LD is endemic in the Canadian province of Nova Scotia (NS), with most regions of the province considered high risk. In 2019, the incidence of LD in NS was the highest in Canada at 85.6 per 100,000 population, which is 12 times the national rate (3).

LD presents in three stages: early localized, early disseminated, and late disseminated LD. Early localized disease occurs within the first few weeks following infection from a tick bite (4). Cutaneous manifestations of early localized infection include erythema migrans, an expanding erythematous rash occurring at the site of the tick bite. Untreated, the spirochetes can disseminate throughout the body progressing to early and late disseminated stages over weeks to months. Early disseminated symptoms may include multiple erythema migrans lesions, meningitis, facial palsy, and carditis. This stage can be accompanied by fatigue, myalgia, arthralgia, and fever. Late disseminated disease often occurs months after initial infection and primarily affects the musculoskeletal system, typically causing monoarticular or oligoarticular arthritis, or less commonly chronic neuroborreliosis. Lyme arthritis is more common in North America compared with cases of LD in Europe (5).

Children under 15 years of age appear to be at higher risk of developing Lyme arthritis compared to other age groups (6). Lyme arthritis can present similarly to other causes of joint pain and swelling in children (e.g., septic arthritis, juvenile idiopathic arthritis [JIA], reactive arthritis, hemarthrosis, mechanical causes). For example, children with Lyme arthritis can present acutely with fever, elevated inflammatory markers, and marked synovial fluid pleocytosis, which overlaps predominantly with the presentation of septic arthritis (7, 8). In contrast, some children with Lyme arthritis present sub-acutely, without associated fever, which would point away from septic arthritis and overlap with the presentation of inflammatory arthritis such as JIA.

Although septic arthritis is overall a rare diagnosis, it is crucial to differentiate it from Lyme arthritis in a timely manner due to differences in treatment, management, and possible complications. First-line treatment for Lyme arthritis is oral antibiotics, while the management of septic arthritis requires prompt surgical intervention, intravenous antibiotics for organisms associated with septic arthritis, and careful monitoring in hospital to mitigate the risk of long-term articular complications including rapid cartilage destruction (9).

Due to the potential catastrophic sequelae of missing a diagnosis of septic arthritis and the delay in obtaining serologic test results for LD, a proportion of children with Lyme arthritis are treated empirically for septic arthritis (10, 11). Therefore, there is a risk that patients with Lyme arthritis may undergo unnecessary investigations, surgical procedures, and hospital admission in addition to receiving inappropriate antibiotic therapy. A recent report from an academic tertiary pediatric hospital in New York found that 10% of children with Lyme arthritis initially underwent surgical treatment for septic arthritis (12). Such surgical interventions can result in additional morbidity and have been shown to increase hospital resource use and costs. In addition, a delay in the diagnosis of Lyme arthritis, including the presence of joint symptoms for ≥6 weeks, has been associated with a higher risk of antibiotic-refractory Lyme arthritis in children, further underpinning the importance of promptly diagnosing Lyme arthritis (13).

The diagnosis of Lyme disease relies on serologic testing using either the standard two-tiered testing (STTT) algorithm where antibodies to *B. burgdorferi* are detected using an enzyme immunoassay (EIA) followed by confirmatory immunoblots measuring IgM or IgG antibodies, or the modified two-tiered testing (MTTT) algorithm where an initial EIA is used to identify positives and a second EIA is used for confirmatory testing. The performance of any serologic testing depends on the stage of infection. Due to Lyme arthritis typically presenting months after exposure, the sensitivity of STTT has been shown to exceed 99% for diagnosing Lyme arthritis (14). A drawback of the STTT algorithm is it often requires sending specimens to a referral laboratory, leading to final result turnaround times of several weeks. The MTTT algorithm offers improved sensitivity and equivalent specificity to the STTT, with a shorter turnaround time for final result; however, it can take up to 3 days because of the requirement for batch testing (15). This timing remains suboptimal for acute clinical decision-making in patients who present with septic arthritis as part of the differential diagnosis. Having an accurate and rapid method for identifying Lyme arthritis would improve patient care by differentiating it from other forms of arthritis and has the potential to reduce the morbidity associated with unnecessary invasive interventions.

The Quidel Sofia Lyme Fluorescent Immunoassay (Sofia) is a rapid serologic assay that can detect IgG and/or IgM antibodies to *B. burgdorferi* in 10 minutes. When used at point of care, this rapid serologic test has the potential to fill an important gap in the diagnostic tools available to clinicians. This study aimed to evaluate whether the Sofia had sufficient sensitivity, specificity, and predictive value to identify children with Lyme arthritis and differentiate them from those with other musculoskeletal presentations including septic and inflammatory arthritis.

## MATERIALS AND METHODS

### Study design

This was a retrospective study using residual serum samples collected from children ≤16 years of age who underwent LD testing as part of clinical care at the IWK Health Centre. Based on patient numbers seen in clinical practice and laboratory storage procedures, we estimated that we would be able to retrieve approximately 50 samples from children diagnosed with Lyme arthritis. Specificity levels with 95% confidence intervals (CI) were calculated for a range of specificities based on a sample size of 50. A priori we determined that 90% specificity (CI 0.78–97) would be an acceptable cutoff. These samples were stored at −20°C at the provincial microbiology laboratory at Nova Scotia Health from 2016 to 2022. The serum samples were de-identified and linked to clinical data through a unique study number. Samples were included if the reason for LD testing was a musculoskeletal sign or symptom (any one of joint pain, joint swelling, or joint stiffness). The clinical diagnosis of the patient (Lyme disease vs other causes of musculoskeletal signs or symptoms), age, and sex were extracted from the medical record. To be included as a Lyme arthritis sample, the patient’s diagnosis had to meet the definition of late disseminated LD as outlined by the Centers for Disease Control and Prevention, which requires a positive EIA result followed by a positive result of at least 5/10 bands present on IgG immunoblot (16).

All sera were initially tested for LD using the Zeus C10/VlsE EIA total antibody (ZEUS ELISA Borrelia VlsE1/pepC10 IgG/IgM). Those with positive results were subsequently tested at the National Microbiology Laboratory in Winnipeg, Manitoba, using the EUROIMMUN Anti-Borrelia burgdorferi US EUROLINE-WB (IgG). If the IgG immunoblot was negative, the serum was tested using the EUROIMMUN Anti-Borrelia EUROLINE-RN-AT-adv (IgM) kits (STTT). Sera that tested negative for LD using the Zeus C10/VlsE EIA were considered negative and subjected to no further testing.

The Sofia was used as per the manufacturer’s instruction. Briefly, 30 μL of thawed sera was added to the test buffer, after which 100 µL was transferred to the center sample well on the lateral flow cassette. Following a 10-minute incubation at room temperature, the cassette was inserted into the Sofia 2 analyzer and read using the “READ NOW” mode. The presence of IgM and IgG antibodies to *B. burgdorferi* was recorded for each specimen. Any discrepant results with the STTT were repeated by both the Sofia and STTT.

The analytical specificity of the Sofia was further evaluated using sera from healthy adults (*n* = 29) and adults with conditions that could potentially cause false-positive results with the STTT, including sera from patients with positive Epstein-Barr virus (EBV) viral capsid antigen (VCA) IgM (*n* = 10), syphilis serology (*n* = 10), or anti-nuclear antibody (ANA) (*n* = 10). The sera from healthy individuals were from a prior serosurvey (17). EBV VCA IgM and ANA testing were carried out using the BioPlex 2200 EBV IgM and BioPlex 2200 ANA screen with MDSS (Bio-Rad Laboratories, Hercules, CA, USA), respectively. Positive syphilis samples were reactive using both the Architect Syphilis TP assay on the Architect i2000SR (Abbott Diagnostics, Lake Forest, IL, USA) and confirmatory testing using the Treponemal Particle Agglutination (TPPA) (Serodia-TP-PA, Fujirebio Inc., Gent, Belgium). The STTT was used to test all samples in the analytical specificity testing.

Clinical data were analyzed using descriptive statistics. The performance of the Sofia was determined using the STTT result as the reference standard. A true positive was defined as having a positive IgG immunoblot using the Health Canada interpretive criteria established by the National Microbiology laboratory where at least 5/10 significant bands were present. A true negative would have negative results using the STTT. The focus of this study was the Sofia IgG result, as it is expected to be positive in individuals with Lyme arthritis, a late manifestation of LD.

Using MedCalc Software Ltd. Diagnostic test evaluation calculator (https://www.medcalc.org/calc/diagnostic_test.php [Version 22.009; accessed 6 August 2023]), we report the accuracy of the Sofia using standard test characteristics: sensitivity, specificity, and likelihood ratios with 95% CI. Positive or negative predictive values were excluded from the analysis, as the samples do not represent the true prevalence of Lyme arthritis in children in NS.

## RESULTS

We retrieved 51 samples from patients who were LD positive with a diagnosis of Lyme arthritis. Approximately the same number of samples (*n* = 55) was retrieved from patients who were LD negative. The median age of the children whose samples were analyzed was 11 years (range 1.6–16); 61 males and 45 females. For the patients who were LD negative, the clinical diagnoses included juvenile idiopathic arthritis (*n* = 21), mechanical joint pain (*n* = 14), arthralgia (*n* = 8), reactive arthritis (*n* = 4), septic arthritis (*n* = 1), and other (*n* = 7).

Serological testing using the Sofia was conducted on all 106 residual serum specimens (Table 1). The Sofia IgG was positive in all 51 LD-positive samples. Of the 55 LD-negative samples, the Sofia IgG was negative in 53 (96.4%) and positive in 2 (3.6%). The two positive samples were from two children with juvenile idiopathic arthritis (a 12-year-old female and a 16-year-old male); both samples tested negative for LD by STTT (including both the EIA and Western blot). The sensitivity and specificity of the Sofia IgG result to identify cases of Lyme arthritis in children were 100% (95% CI of 93.0%–100%) and 96.4% (95% CI: 87.5%–99.6%), respectively. This translates into a positive likelihood ratio (LR) of 27.5 (95% CI 7–107) and a negative LR of 0.00 (95% LR 0.00–0.15).

**TABLE 1:** Sofia IgG results in children with musculoskeletal complaints tested for LD

	Standard two-tier test		Total
Sofia IgG	Positive	Negative	
Positive	51	2	53
Negative	0	53	53
Total	51	55	106

Although the Sofia IgM result was not the focus of our study, we found the Sofia IgM to be positive in 37/51 (72.5%) LD-positive samples and in 9/55 (16.4%) LD-negative samples. Of the 9 LD-negative samples, there was a sufficient quantity of serum in 8 for additional testing; all of which were confirmed to be negative on IgM immunoblot, suggesting true false-positive reactions.

Given the known cross-reactivity that can occur with the STTT in certain infections and syndromes, the analytical specificity of the Sofia was further evaluated using a specificity panel containing 59 residual sera that were LD negative by STTT (Table 2). This included healthy adult patients and patients positive for either EBV, syphilis, or ANA. Of the 29 healthy patients, 16 samples were negative for both IgM and IgG, 1 sample was IgM-/IgG+, and 12 samples were IgM+/IgG-. Of the 30 samples with positive ANA or infectious serology, 16 were negative for both IgM and IgG, 5 IgM-/IgG+ (4 ANA, 1 EBV), 7 IgM+/IgG- (2 EBV, 5 Syphilis), and 2 IgM+/IgG+ (2 EBV). In total, there were 8 samples that tested positive for IgG and 21 for IgM on the Sofia, all of which tested negative on the corresponding immunoblot (Table 2).

**TABLE 2:** Sofia specificity assessed by IgM and IgG results in samples negative for LD by STTT

Sofia result	Sample type				Total
	Healthy patients	ANA positive	EBV VCA positive	Syphilis positive	
IgM neg/IgG neg	16	6	5	5	32
IgM neg/IgG pos	1	4	1	0	6
IgM pos/IgG neg	12	0	2	5	19
IgM pos/IgG pos	0	0	2	0	2
Total	29	10	10	10	59

## DISCUSSION

Our study is the first to evaluate the diagnostic performance of the Sofia in a population of children who underwent Lyme testing for one or more of joint pain, joint swelling, or joint stiffness in a Lyme-endemic region. The Sofia IgG performed well with high sensitivity and specificity as a rapid test for Lyme arthritis when compared to the reference standard, the STTT algorithm. The positive and negative LRs were 27.5 and 0.00, respectively. Any test with an LR ratio above 10 or below 0.1 is considered a “strong test to rule in and rule out diagnoses respectively,” suggesting that the Sofia IgG would be beneficial as a point-of-care test used in conjunction with clinical and standard laboratory tests when diagnosing pediatric Lyme arthritis (18). The analytical specificity testing using selected adult patient samples, however, showed that the Sofia was prone to false positives; 36% of samples were IgM positive and 17% were IgG positive. This emphasizes the need to consider the specific context in which the Sofia is studied or implemented in clinical care.

To our knowledge, our study is the first to evaluate the performance of the Sofia focusing on a clinically characterized population and to include a specificity analysis. In the one comparable study examining the validity of the Sofia, Lewandrowski et al. evaluated its performance as a first-tier test against the Zeus ELISA, their predicate first-tier assay, using archived clinical samples (19). The Sofia was considered positive if either the IgM or the IgG was positive. They found that the Sofia performed well as the first step in an STTT algorithm for LD with a positive percent agreement (PPA) of 96.2% and a negative percent agreement (NPA) of 100%. The current study, which used the same STTT as the reference method, demonstrated almost identical performance characteristics with a sensitivity and specificity of 100% and 96.4%, respectively, when the Sofia results were limited to IgG. Both validation studies, therefore, suggest that the Sofia performs well when compared to the reference standard of the STTT. In contrast to the Lewandrowski study, which lacked clinical information corresponding with the sera tested, our study allows us to hypothesize a specific clinical scenario in which the Sofia IgG could be used to direct care.

Lyme arthritis is a late stage of infection in which the IgG antibodies will invariably be present, making serologic testing using the two-tier approach the gold standard for its diagnosis. As highlighted in the Infectious Disease Society of America guidelines, an alternative cause for the patient’s presentation should be considered if only IgM antibodies are present (20). In the current study, 14.5% (8/55) of sera from non-Lyme patients had detectable IgM in the absence of IgG, representing false-positive serologic results (none of these patients had symptoms consistent with early LD on chart review). Similarly, in the analytical specificity panel, 21/59 (35.6%) had positive Sofia IgM results and 8/59 (13.5%) had positive Sofia IgG results, all of which were negative by STTT. Previous studies have demonstrated cross-reactive results in serologic tests for LD in a variety of infectious syndromes (21). The Sofia IgM appears to be slightly more prone to false positives/cross-reactivity compared to the Sofia IgG. We propose that in the clinical scenario of evaluating a child with arthritis in a Lyme-endemic region, when using the Sofia, only the IgG result should be displayed, as this is a programmable feature of the Sofia analyzer.

A false-positive IgG on the Sofia could lead to unnecessary treatment with antibiotics and misattribution of a clinical presentation of LD while waiting for the STTT or MTTT result. A false-positive Sofia IgG was found in two patients in the non-Lyme disease cohort, both with JIA. In the case of JIA, there would be little risk in waiting for the results from a two-tiered test. If the patient was treated as having Lyme arthritis, it would mean treatment with unnecessary antibiotics and a delay in initiating appropriate treatment for JIA. The consequences of this are unlikely to impact the long-term outcome of these patients. In contrast, basing clinical decision-making solely on a positive Sofia IgG in a child who actually has septic arthritis could have catastrophic consequences. This emphasizes the importance of considering the entire clinical picture if the Sofia IgG is to be used in this clinical context.

Several studies have assessed clinical prediction tools to gauge the risk for septic arthritis in children presenting with monoarthritis in Lyme-endemic regions. A retrospective study found that an erythrocyte sedimentation rate ≥40 mm/hour and an absolute neutrophil count of ≥10 × 10^3^ cells/mm^3^ were both independent clinical predictors of septic arthritis, with both features identifying all patients with septic arthritis of the knee in a Lyme-endemic region (7). This tool was recently validated externally in a separate study and was found to perform well prospectively in a multi-center trial including 457 children with knee monoarthritis (22). Another study identified different clinical factors serving as independent predictors of septic arthritis including a history of fever, pain with short arc motion, a C-reactive protein level ≥40 g/L, and age younger than 2 years (23). Another study found that refusal to weight bear was the strongest predictor of septic arthritis (24). The Sofia IgG, used in conjunction with clinical variables (e.g., ability to weight bear, reassuring laboratory values), could strengthen the power of existing clinical prediction tools in identifying children who are at low risk of septic arthritis. This approach, which requires prospective validation and knowledge of the background prevalence rate of seropositivity in a given population, could allow clinicians to be more confident in making decisions around the need for invasive investigations, interventions, and treatments, thereby also reducing unnecessary patient morbidity and health care expenditures.

The primary limitation of this study is the inclusion of only one case of septic arthritis. The Sofia IgG was negative for this sample. Septic arthritis was diagnosed in only 19 (3%) of 673 children presenting to two urban, tertiary care emergency departments with knee monoarthritis in a Lyme-endemic area over 12 years (22). Thus, although we knew septic arthritis samples would be limited, we had anticipated more than just a single sample over 6 years. Despite this, we feel our study provides a strong rationale to study the role of the Sofia IgG in children presenting with joint pain and/or swelling in Lyme-endemic areas. Another potential limitation is the use of frozen residual sera. Although freeze-thaws can potentially affect the performance of testing, there are data on other serologic tests that suggest that antibody concentrations for vaccine preventable disease are stable for up to 10 freeze-thaw cycles (25). Finally, as more of the population acquires Lyme disease in our region, seropositivity will increase. Studies have shown that antibody reactivity, both of IgG and of IgM, can remain for years to even decades following LD infection (26, 27). This renders presently available serological tests, including the Sofia, of limited value in diagnosing LD, highlighting the need for the development of novel testing approaches to distinguish between active and past infection with LD.

### Conclusion

When compared to the reference standard, the STTT algorithm, the Sofia IgG performed well in diagnosing and ruling out Lyme arthritis in a population of children with musculoskeletal symptoms in a region with a high prevalence of LD. As a point-of-care test, the Sofia IgG has the potential to assist in clinical decision-making. Given our findings, the next step will be to implement prospective quality initiatives in our emergency department to test the ongoing performance of the Sofia IgG. This approach will limit selection bias and determine more precisely how the Sofia IgG, in addition to other clinical and laboratory values, could impact clinical decision-making and outcomes for this patient population.
